# Acute oxygenation changes on ischemic foot of a novel intermittent pneumatic compression device and of an existing sequential device in severe peripheral arterial disease

**DOI:** 10.1186/1471-2261-14-40

**Published:** 2014-03-31

**Authors:** Fabio Manfredini, Anna Maria Malagoni, Michele Felisatti, Simona Mandini, Nicola Lamberti, Roberto Manfredini, Francesco Mascoli, Nino Basaglia, Paolo Zamboni

**Affiliations:** 1 Department of Rehabilitation Medicine, S. Anna Hospital University, Ferrara, Italy; 2 Vascular Diseases Center, University of Ferrara, Ferrara, Italy; 3 Program Pathophysiology of Vascular Peripheral System and Day Surgery, S. Anna Hospital University, Ferrara, Italy; 4 Clinica Medica, Department of Medical Sciences, University of Ferrara, Ferrara, Italy

**Keywords:** Intermittent pneumatic compression devices, Near-infrared spectroscopy, Peripheral vascular disease, Perfusion, Critical limb ischemia

## Abstract

**Background:**

Intermittent pneumatic compression (IPC) improves haemodynamics in peripheral arterial disease (PAD), but its effects on foot perfusion were scarcely studied. In severe PAD patients we measured the foot oxygenation changes evoked by a novel intermittent IPC device (GP), haemodynamics and compliance to the treatment. Reference values were obtained by a sequential foot-calf device (SFC).

**Methods:**

Twenty ischemic limbs (Ankle-Brachial Index = 0.5 ± 0.2) of 12 PAD patients (7 male, age: 74.5 ± 10.8 y) with an interval of 48 ± 2 hours received a 35 minute treatment in supine position with two IPC devices: i) a Gradient Pump (GP), which slowly inflates a single thigh special sleeve and ii) an SFC (ArtAssist^®^, ACI Medical, San Marcos, CA, USA), which rapidly inflates two foot-calf sleeves. Main outcome measure: changes of oxygenated haemoglobin at foot (HbO_2foot_) by continuous near-infrared spectroscopy recording and quantified as area-under-curve (AUC) for periods of 5 minutes. Other measures: haemodynamics by echo-colour Doppler (time average velocity (TAV) and blood flow (BF) in the popliteal artery and in the femoral vein), patient compliance by a properly developed form.

**Results:**

All patients completed the treatment with GP, 9 with SFC. HbO_2foot_ during the working phase, considered as average value of the 5 minutes periods, increased with GP (AUC 458 ± 600 to 1216 ± 280) and decreased with SFC (AUC 231 ± 946 to −1088 ± 346), significantly for most periods (P < 0.05). The GP treatment was associated to significant haemodynamic changes from baseline to end of the treatment (TAV = 10.2 ± 3.3 to 13.5 ± 5.5 cm/sec, P = 0.004; BF = 452.0 ± 187.2 to 607.9 ± 237.8 ml/sec, P = 0.0001), not observed with SFC (TAV = 11.2 ± 3.4 to 11.8 ± 4.3 cm/sec; BF = 513.8 ± 203.7 to 505.9 ± 166.5 ml/min, P = n.s.). GP obtained a higher score of patient compliance (P < 0.0001).

**Conclusions:**

A novel IPC thigh device, unlike a traditional SFC device, increased foot oxygenation in severe PAD, together with favourable haemodynamic response and high compliance to the treatment under the present experimental conditions.

## Background

Intermittent pneumatic compression (IPC) devices, used for prophylaxis and treatment of venous and lymphatic pathologies [1,2], have become of potential interest for arterial pathologies. The wide spectrum of haemodynamic effects of IPC on shear stress, vascular tone and arterial-venous pressure gradient increase [3-7] make IPC devices effective in treating arterial pathologies at intermediate disease stages [8-13], at severe stages even in presence of tissue lesions [14-18], or post-operatively to reduce the risk of bypass graft thrombosis [19]. However, not always successful effects of these devices on foot perfusion have been also reported [20,21]. IPC devices include a pneumatic impulse generator and inflatable plastic units surrounding a selected area of the lower limb (mainly the calf and foot or both). They differ in terms of the size and seat of the sleeve and in the patterns of compression. Sequential compression devices are haemodynamically superior [9,22-24].

The optimal IPC device should be comfortable, easy to use, and effective in a short treatment time to improve compliance. With this in mind, a novel intermittent thigh compression device for patients with lower limb ischaemia [25], called Gradient Pump (GP), was recently designed on a different haemodynamic concept and tested to optimize the therapeutic cycle.

The aim of this study is to describe the acute effects of this original device on foot perfusion by Near-Infrared Spectroscopy (NIRS), haemodynamic parameters, and patient compliance in subjects with severe peripheral arterial disease (PAD). These results were compared with those obtained on the same patients using a sequential foot-calf compression device (SFC) available on the market.

## Methods

### Subjects

Twelve patients (M = 7, age = 74.5 ± 10.8 y) including 20 diseased limbs affected by PAD at Fontaine stage IIb-III-IV (Ankle Brachial Index = 0.5 ± 0.2) were measured after a preliminary clinical and instrumental screening at the Vascular Surgery Department. All patients had episodes of critical limb ischemia (CLI) over the past 2 months, with 9 patients (for a total of 14 legs) experiencing symptoms in the week prior to the evaluations (Ankle Brachial Index ≤ 0.4 and/or ischemic ulcers/skin necrosis). The exclusion criteria were: chronic venous insufficiency of the leg, recent deep vein thrombosis (< 6 months), extensive infected ulceration, and severe congestive heart failure (≥ NYHA 3). All participants gave their written informed consent. The study was approved by the ethical committee of Ferrara (Italy). The characteristics of the participants are reported in Table 1.

**Table 1:** Characteristics of study subjects

**Subjects/legs, n**	**12/20**
Age (y)	74.5 ± 10.8
Sex (M/F)	7/5
BMI	26.7 ± 12.4
PAD II B, legs, n (%)	6 (30%)
PAD III, legs, n (%)	12 (60%)
PAD IV, legs, n (%)	2 (10%)
*Legs lesion location, n (%)*	
Aorto-iliac	2 (10%)
Femoral	20 (100%)
Popliteal	3 (15%)
Infra-pop	10 (50%)
*Risk factors and comorbidites, n (%)*	
Familiarity for vascular diseases	4 (33%)
Smoke	8 (67%)
Hypercholesterolemia	7 (58%)
Hypertension	10 (83%)
Diabetes	5 (42%)
Heart diseases	2 (17%)
End stage renal disease	2 (17%)
Stroke	1 (8%)

*Abbreviations*: *BMI* Body Mass Index, *PAD* Peripheral Arterial Disease (stages are according to Leriche-Fontaine classification).

### Intermittent compression devices

The GP [25] consists of an inflatable sleeve having a length of 50–80 cm and a width of 13 cm, to be positioned over the mid thigh with a rigid element inside (length: 9 cm, width: 10 cm) to selectively improve the compressive effect on the femoral vein. The inflatable cuff is connected to a compressor that produces variable levels of pressure set by a manual regulator. The device includes a manual electromechanical timer to produce periodic sequences of pressure and the modification and control of the working/resting cycles. The GP was set to deliver an operative cycle of a progressive inflation with a compression phase lasting 20 seconds and a later decompression phase lasting 40 seconds (1 cycle/minute).

The intermittent sequential foot-calf compression device (SFC) (ArtAssist ACI Medical, LLC San Marcos, CA, USA), available on the market, consists of a compression sleeve positioned at the foot and at the calf and supplies a high sequential pressure of 120 mmHg first at the foot and after 3 seconds at the calf, with 17 seconds of virtually no pressure (3 cycles/minute).

### Treatment

The effects of the GP and SFC were measured in the same subject in the supine position on two different days with an interval of 48 ± 2 hours. The devices were alternated so each treatment was used in sequence, with an equal number of times as first treatment. Each device was applied after a 10-minute rest in the supine position. An intermittent treatment was proposed for GP, based on 5 minutes of work (device on) followed by 5 minutes of rest (device off), for a total of 20 minutes of work and 35 minutes of total treatment at a pressure corresponding to the systolic blood pressure minus 20 mmHg with a maximum value of 120 mmHg. The SFC was applied for 2 consecutive hours, considering an average duration of previously published treatment indications [10,12,3,18]. The difference in device appearance made it impossible to fully blind the patients. However, the patients did not know which was under study. The operators were not blinded to the treatments.

### Outcome measures

**Figure 1:**
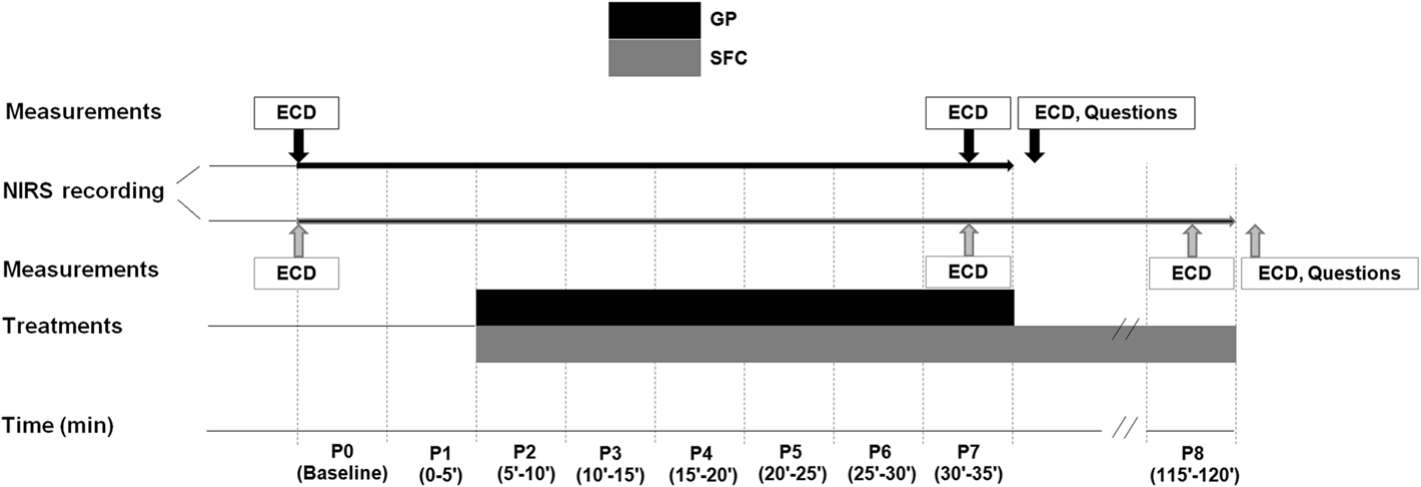
**A schematic representation of the experiment.** Legend to Figure 1: GP, Gradient Pump Device; SFC, sequential foot-calf device; NIRS, near-infrared spectroscopy; ECD, echo-colour Doppler.

A schematic description of the study design is shown in Figure 1.

#### ***Foot perfusion measurements by near-infrared spectroscopy***

The foot perfusion was measured with a continuous wave system (Oxymon-MK III, Artinis Medical Systems, Netherlands) providing measures of changes in oxy (O_2_Hb) and deoxyhaemoglobin (HHb) concentrations. The instrument consists of two channels (two equal-pulsed light sources, two avalanche photodiode detectors and ambient light protection) using intensity-modulated light at a 1 Hz frequency and laser diodes at three wavelengths (905, 850 and 770 nm) corresponding to high absorption of O_2_Hb and HHb with an auto-sensor operating at 110–240 V (approximately 40 W). The sum of the O_2_Hb and HHb, total haemoglobin (tHb), and the difference between O_2_Hb and HHb were calculated. The sensors were positioned at the dorsum of the foot as previously performed by other Authors [26-28], crossing the first intermetatarsal space, marking the position of probes on the skin using a dermographic pen to ensure that the same area was studied during both treatments. The measurements were performed continuously for 5 minutes before the treatment to obtain baseline values and also during the whole treatment duration. Every five minutes, a marker was included during the phase of recording to facilitate the NIRS trace analysis.

The semiquantitative data obtained as a function of time were extracted by the software provided by the NIRS system manufacturer (Oxysoft 2.47) and were transferred to an electronic spreadsheet (Microsoft Excel 2007). After normalization to zero, the data were analysed with statistical software (Medcalc 12.2.1.0, Medcalc-Software, Mariakerke, Belgium) to determine the area under the curve (AUC) to quantify the individual variations in HbO_2AUC_ and tHb_AUC_, as previously reported [29].

#### ***Echo-colour Doppler measurements***

The echo-colour Doppler measurements were performed before, after 30 minutes of treatment during an operative cycle, and at the end of the treatment (device off). During the operative cycle, the phase of compression (C phase), and phase of decompression (D phase) were evaluated. For the GP only, due to its long duration, the phase of compression was divided into two sub-phases: the early (pushing) phase, (C_p_ phase, 5 seconds), and the phase of full (closing) compression (C_c_ phase, 15 seconds). A LogiQ S6 (General Electrics, Fairfield, CT, USA) was used.

The time average velocity (TAV) and the blood flow (BF) in the popliteal artery and in the femoral vein at the inguinal level were assessed. If the popliteal artery was occluded, haemodynamics were recorded in the Hunterian or gastrocnemius artery. To insonate vessels at the same point, the positioning point of the probe was outlined over the patient skin. All of the assessments were performed by the same operator using a 7.5 MHz linear array probe in the longitudinal aspect. Doppler sample volume was placed in the middle of the lumen, with the angle of incidence of the ultrasonic beam at 45°-60° (parallel to the flow direction). TAV was obtained by marking the flow spectrum on the screen of at least five cycles, and using automatic calculation by the software of the ultrasound machine. To obtain the vessel diameter measurement, two cursor points were placed on the lumen edges of the near and far vessel walls, perpendicular to the vessel position on the screen. The BF value was automatically calculated from these two measurements.

#### ***Patient’s compliance***

A form including 10 closed-ended questions was developed to evaluate the devices in terms of symptoms, side-effects, satisfaction, ease of use, and tolerance (Table 2). A dichotomic yes/no response format was used. A score of 1 or 0 was assigned to each question for a favourable or unfavourable judgment, respectively. A higher total score corresponded to increased patient compliance, and vice versa. Patients were interviewed by the same operator after each treatment period.

**Table 2:** The questions form used to evaluate patient compliance with treatments and the scores assigned

	**Yes**	**No**
1) Did the treatment get pain in your leg/foot?	0	1
2) Did you feel worsening of pain in your leg/foot during the treatment?	0	1
3) Did you need to interrupt the treatment because of pain?	0	1
4) Did you feel relief from pain in your leg/foot during the treatment?	1	0
5) Did you experience pain in your leg/foot or worsening of pain after the treatment?	0	1
6) Did you feel discomfort/pain at the site of the sleeve?	0	1
7) Is the device easy to use?	1	0
8) Was the duration of the treatment acceptable?	1	0
9) Would you be willing to continue the treatment at home for 7 days?	1	0
10) Would you recommend the use of the device to somebody with your problem?	1	0

Questions 2 and 4 considered the presence of ischemic pain before the treatment.

### Statistical analysis

The continuous variables are expressed as mean ± standard deviation. The normal distribution of data was verified by the Kolmogorov-Smirnov test.

Since the baseline values were taken for a 5-minute period, the effect on foot perfusion of each device (intra-comparison analysis) was evaluated by comparing the AUC values of the NIRS parameters of each subsequent 5-minute treatment period with the 5-minute baseline value, using the paired Student’s T-test. For each device, the intra-comparison analysis of the haemodynamic parameters was performed by comparing the baseline haemodynamic measurements and those collected at 30 minutes and at the end of the treatment using the paired Student’s T-test. The compliance form scores from the two devices were compared by the unpaired Student’s T-test. A p-value ≤0.05 was considered statistically significant. Medcalc 12.2.1.0 software program (Medcalc-Software, Mariakerke, Belgium) was used.

## Results

All of the patients completed the GP treatment period. Three patients discontinued the treatment with SFC because of pain in the compression sleeve. Two of them stopped the treatment after 35 minutes, one patient stopped after 10 minutes. No significant changes in systolic (SBP) and diastolic (DBP) blood pressure were observed following treatment with the two devices (GP: baseline SBP = 139 ± 23, DBP = 75 ± 11; end SBP = 143 ± 11; DBP = 75 ± 13; SFC baseline SBP = 140 ± 19, DBP = 74 ± 10; end SBP = 156 ± 24; DBP = 81 ± 9). The average settled pressure of inflation with GP was 112 ± 12 mmHg.

### Foot perfusion

**Figure 2:**
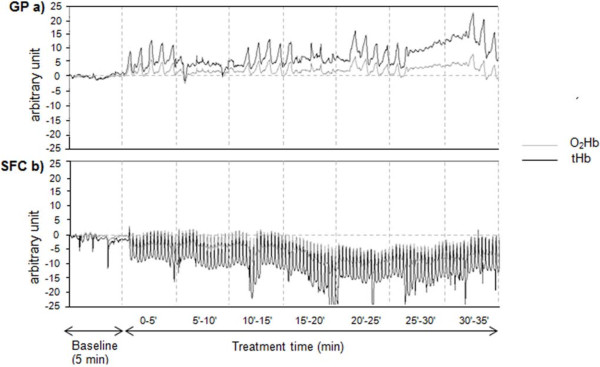
**Monitoring of foot oxygenation changes by NIRS with the two devices in the same subject.** Legend to Figure 2: **a)** GP, Gradient Pump device; **b)** SFC, sequential foot-calf device; HbO_2,_ oxygenated haemoglobin; tHb, total haemoglobin.

One patient (2 diseased legs) was excluded from the intra-SFC analysis because the treatment with SFC was stopped early. Seven 5-minute periods of NIRS traces were recorded during the 35 minutes of treatment for both devices (Figure 2).

For the SFC, also the last 5-minute period (8th period, from 115 to 120 minutes) recorded prior to the end of the 2-hour treatment was considered for the intra-comparison analysis, except for 3 patients (4 diseased legs) who discontinued the treatment. For the GP, the 1st, 3rd, 5th and 7th periods correspond to the intermittent compression phases.

**Figure 3:**
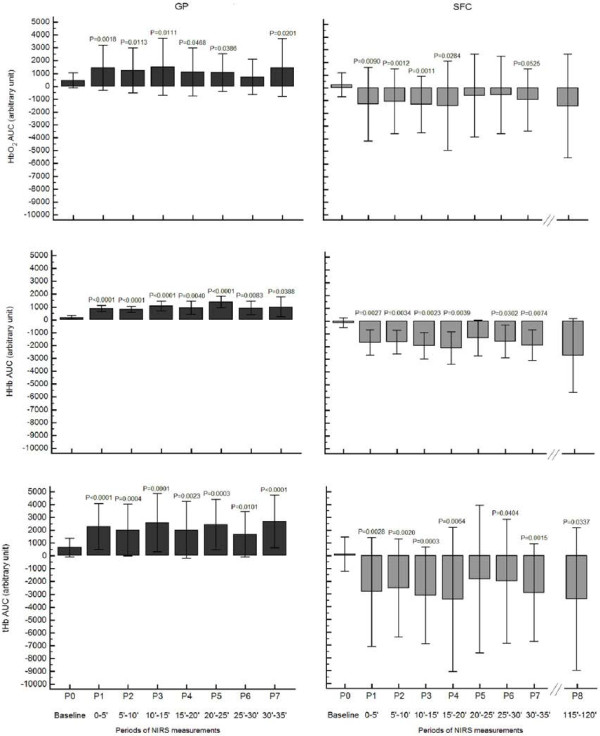
**HbO**_**2AUC**_**, HHb**_**AUC**_**, and tHb**_**AUC **_**changes during the treatment with the two devices.** Legend to Figure 3: GP, Gradient Pump device; SFC, sequential foot-calf device; HbO_2AUC_, area under curve of oxyhaemoglobin; HHb_AUC_, area under curve of deoxyhaemoglobin; tHb_AUC_, area under curve of total haemoglobin; P1-8, 5’ periods of measurements during the treatment. Data refer to 12 patients/20 diseased legs for GP device, and 11 patients/18 diseased legs for SFC device.

For the GP, a significant higher trend of the NIRS parameters in respect to baseline for most of the 5-minute periods was recorded, differently from the SFC that showed a lower trend in respect to baseline during the whole treatment (Figure 3). The trend for SFC was not different following a prolonged treatment, with HbO_2AUC_ and tHb_AUC_ values between 115–120 minutes slightly lower than at 30–35 minutes.

**Figure 4:**
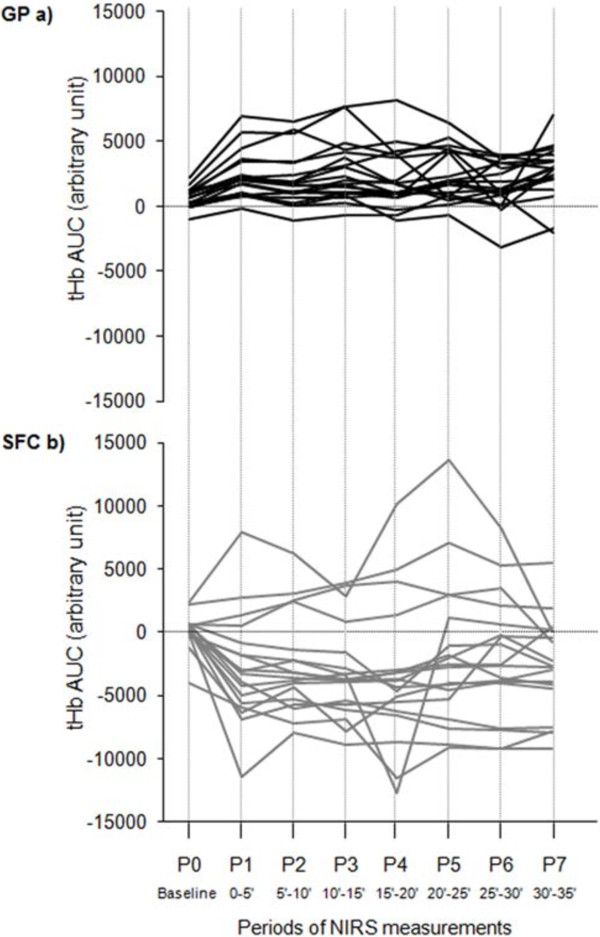
**tHb**_**AUC **_**changes for each single leg under study during the treatment with the two devices.** Legend to Figure 4: **a)** GP, Gradient Pump device; **b)** SFC, sequential foot-calf device; tHb_AUC_, area under curve of total haemoglobin. The continuous lines connect tHb_AUC_ values recorded each 5-minute period during the treatment (P1-7) starting from baseline (P0). Data refer to 12 patients/20 diseased legs for GP device, and 11 patients/18 diseased legs for SFC device.

The tHb_AUC_ trend for each single leg under study during the two treatments showed a more heterogeneous response during SFC treatment (Figure 4).

### Echo-colour Doppler measurements

One patient (2 diseased legs) was excluded from the SFC analysis because the treatment was stopped early. In a few patients_,_ due to the involuntary movements of the leg during the C phase at 30 minutes, it was not possible to accurately collect the data at the arterial side for both devices (GP: n = 3 patients/5 legs; SFC: n = 5 patients/7 legs). Missing data were assumed not different from the D phase. On the contrary all data were correctly registered at the venous side in all phases.

During the C_p_ and D phases at the femoral vein, the GP treatment was followed by a significant increase in TAV (94% and 53%, respectively) and BF (112% and 62%) after 30 minutes, while in C_c_-phase, a slight but significant decrease in BF was recorded (−15%). Significant increases in both parameters were recorded at the end of the treatment (36% and 44%). At the arterial side, a significant improvement in TAV and BF was observed at the end of treatment (66% and 71%) (Table 3).

**Table 3:** The hemodynamic parameters measured by echo-colour Doppler with the two devices under study

		**TAV**_**FV**_	**P value**	**BF**_**FV**_	**P value**	**TAV**_**PA**_	**P value**	**BF**_**PA**_	**P value**
		_**(cm/sec)**_	**TAV**_**FV**_	_**(ml/min)**_	**BF**_**FV**_	_**(cm/sec)**_	**TAV**_**PA**_	_**(ml/min)**_	**BF**_**PA**_
			**Baseline vs phases**		**Baseline vs phases**		**Baseline vs phases**		**Baseline vs phases**
**GP**	Baseline	10.2 ± 3.3		452.0 ± 187.2		13.8 ± 6.9		96.8 ± 73.6	
	C_P_-30’	18.6 ± 6.7	<0.0001	799.9 ± 351.2	0.0004	14.1 ± 6.8	n.s.	103.4 ± 92.1	n.s.
	Cc-30’	8.4 ± 3.8	n.s	343.4 ± 209.3	0.03	13.0 ± 7.2	n.s.	93.1 ± 75.3	n.s.
	D-30’	15.1 ± 6.1	0.0006	632.2 ± 291.2	0.01	14.3 ± 16.6	n.s.	127.3 ± 97.5	n.s.
	End	13.5 ± 5.5	0.004	607.9 ± 237.8	0.0001	16.6 ± 5.7	0.04	154.5 ± 160.5	0.04
**SFC**	Baseline	11.2 ± 3.4		513.8 ± 203.7		15.5 ± 7.9		110.4 ± 81.0	
	C-30’	25.9 ± 7.9	<0.0001	1139.8 ± 269.5	<0.0001	18.6 ± 9.2	n.s.	137.0 ± 79.0	n.s.
	D-30’	10.7 ± 4.7	n.s.	421.7 ± 139.2	0.05	19.0 ± 9.1	n.s.	136.0 ± 82.1	n.s.
	End	11.8 ± 4.3	n.s.	505.9 ± 166.5	n.s.	15.1 ± 6.8	n.s.	110.6 ± 66.3	n.s.

Parameters were measured during the different phases of an operative cycle at baseline, after 30 minutes, and at the end of the treatment.

*Abbreviations*: *GP* Gradient Pump device, *SFC* sequential foot-calf device, *TAV* Time Averaged Velocity, *BF* Blood Flow, *FV* Femoral Vein, *PA* Popliteal artery.

For GP device: Cp, early (pushing) compression phase, and Cc, full (closing) compression phase; D, Decompression phase. For SFC device: C, Compression phase; D, Decompression phase.

Data refer to 12 patients/20 diseased legs for GP device and 11 patients/18 diseased legs for SFC device.

With the SFC, both parameters collected at the femoral vein at 30 minutes resulted in significant differences during the C phase as compared to baseline (147%), but not during the D phase or at the end of the treatment. As for the arterial side, for both parameters a not significant increase was recorded during C phase and D phase at 30 minutes. No changes were recorded for any parameters at the end of the treatment (Table 3).

### Compliance

All patients answered to all questions form. The GP treatment was better tolerated with respect to symptom relief, side effects, satisfaction, ease of use, and tolerance (GP = 9.5 ± 0.7 vs SFC = 5.7 ± 1.8, P < 0.0001).

## Discussion

The study shows that a novel IPC device, based on a different haemodynamic concept and administered with an interval protocol, significantly improved the foot perfusion in the ischemic legs of PAD patients. The same effect was not observed for a traditional sequential foot-calf device tested in the same group of patients. Similarly, positive haemodynamic changes in the veins and arteries of the legs were observed mainly with the GP device, as well as more positive feedback in terms of symptom relief and satisfaction. Previous studies aimed to elucidate the most effective devices to treat PAD patients, has shown that sequential compression devices, particularly the foot-calf devices, were more effective than single compression devices for the foot or calf [9,22-24]. To our knowledge, only one study evaluated the efficacy of a thigh IPC device, and this study promoted the device’s applicability [30]. The novel thigh device was conceived for patients with impaired foot perfusion at rest and CLI, therefore the increased oxygenation of the ischemic foot is the therapeutic target. To evoke a favourable distal haemodynamic effect the proximal inflation at the thigh was preferred in order to create a cyclic squeezing at the femoral vein and generate a pressure gradient to favour the emptying of the distal veins in the decompression phase and the drainage of the congested foot. The present study aimed therefore to evaluate the effects on the microcirculatory unit of the foot as well on the venous and the arterial flow of GP. In order to better evaluate the extent of the haemodynamic response of the device, the effects of an effective sequential IPC device were also measured on the same patients. In previous studies, the foot perfusion was evaluated by laser Doppler flowmetry and transcutaneous oxygen pressure [7,15,16,21]. In our study the NIRS technique was considered optimal for this purpose, for its capacity to detect the local changes of blood flow and the degree of oxygenation below the sensor. Among the NIRS parameters, HHb has been previously considered the parameter better reflecting the dynamic balance between oxygen delivery and oxygen utilization during dynamic exercise, and less influenced by the changes of blood volume [31,32]. In our study we analysed all the NIRS parameters measured by the device but we mainly discuss two of them: HbO_2_ or the amount of Hb with oxygen in the tissue under the sensor, to define the degree of oxygen available in the foot, and tHb or the total blood volume under the sensor.

During the GP treatment, we observed a favourable increase of total haemoglobin as compared to the baseline, corresponding to an increase of blood volume below the sensors for almost all legs (Figure 3), with a concomitant increase of the oxygenated haemoglobin. Following use of the SFC device, we observed a decreased oxygenation at foot with a mean reduction in blood flow, even if the analysis of the single legs (Figure 4) showed and heterogeneous response with few legs improving the blood flow. This effect might be influenced by the phase of compression of the sleeve at the foot in SFC, as previously observed [19,20], avoided in GP device by performing a proximal selective compression at thigh, and by the different degree of compression pressures administered by the devices. SFC, as most of the devices on the market, has a rapid inflation and fixed pressure. The GP, in addition to producing a gradual attainment of the peak pressure, delivers a customizable inflation pressure through a manual regulator to maintain it below the systemic systolic pressure. This approach avoids the risk of transitory arterial occlusion that would potentially create a further reduction of the inflow and a worsening of the hypoxic condition. The administration of an intervalled intermittent pneumatic compression by a manual electromechanical timer enabling a customization of the working and resting phase duration, aims to maintain a balance between oxygen supply and oxygen extraction considering that for the majority of PAD patients previously tested, the HbO_2_ values remain higher than baseline during the off phase of the therapeutic cycle here proposed (personal observation). It has been postulated that one mechanism to explain the changes in arterial leg inflow with IPC is the action on venous pumps [22,30]. In our study, analysing the different phases of a single operative cycle at the venous side, we observed that the two devices work according to different haemodynamic approaches, confirming our original concept. The SFC increases the venous outflow by a double propulsive thrust from the bottom, with a strong action during the compression phase, whereas the GP generates a pressure gradient from the top exerting weaker and more gradual compression, with a therapeutic action during the decompression phase. Our findings, not confirming the increased inflow previously assessed by Doppler ultrasound under the same SFC stimulus [10,16,23], are probably influenced by the different condition of measurements we used and for this reasons we abstained from a inter-comparison analysis between the two devices. Rather than the sitting position, we chose the supine position for different reasons. From the haemodynamic side we excluded the unfavourable action of gravity on the venous return to favour the venous pump. From the clinical side we aimed to test the efficacy of GP in any type of patients including those unable to sit being restricted to bed (e.g. traumatized, severe brain damaged patients) even with leg ulcers. The improved perfusion, relevant for patients with CLI, might in fact represent a crucial factor to favour the repair of trophic lesions, as anecdotally observed in some of the patients included in our rehabilitation program [33].

The evaluation of patient compliance showed a positive result for GP, better tolerated overall than the SFC. We interpret this result along with the lack of pain during treatment, the ability to apply the sleeve at the foot in the presence of ulcers or partial amputation, and the ease of use. The treatment duration plays a role in the adherence to the treatment. With the GP we aimed to limit the treatment to the minimum effective duration, to our knowledge the shortest of all those proposed in literature for the different devices.

### Limitations

Several limitations affect the present study: low sample size, subjects with different disease severity, even if they all experienced pain at rest, measurements taken by operators not blinded to the treatment, and only in the supine position. We are aware that the SFC, an effective device with favourable haemodynamic effects documented by echo-colour Doppler measurements, was tested under not optimal conditions [34]. However, even the supine position, that we considered useful to evaluate the targeted effect of GP on the ischemic foot, is considered an acceptable procedure [35]. Data might be affected by inter-day variability of the haemodynamic conditions. This variability in baseline measurements repeated on the same limbs was not tested, however, for each subject, data were compared to the baseline values. Leg vascular conductance before, during, and after treatment was not calculated, and measurements of toe pressure or transcutaneous oximetry (TcPO_2_), useful to better define the severity of the disease of the subjects, was not assessed.

Some limitations may derive from the NIRS technique, for the limited region evaluated, and the related measurements, that might be affected by the patients’ movement during prolonged treatment. The positioning of the probes at the dorsum of the foot was previously performed by other authors [26-28], even with moderate reliability according to Ubbink and Koopman [27]. We are also aware that the SFC, an effective device with documented favourable haemodynamic effects, was tested under not optimal conditions otherwise useful to evaluate the targeted effect of GP on the ischemic foot.

## Conclusions

The preliminary data presented show that an IPC device designed according to a novel compression concept, carried out at individualized pressure with an interval cycle, induced increased foot perfusion and haemodynamic changes in patients with advanced PAD who well tolerated the treatment. Similar results were not observed using a traditional SFC device under the present experimental conditions. The data need to be confirmed with a large number of patients homogenized on the basis of the severity of disease and acuity of symptoms, and also considering different experimental conditions as the sitting position. The mechanism of action of the device needs to be clearly studied. In addition, large-scale randomized trials in patients with CLI and in populations with ischemic trophic lesions will allow better evaluation of the clinical efficacy of the novel device.

## Abbreviations

AUC: Area under curve; BF: Blood flow; CLI: Critical limb ischemia; C phase: Compression phase; Cc phase: Full (closing) compression; Cp phase: Early (pushing) compression phase; D phase: Decompression phase; HHb: Deoxyhaemoglobin; GP: Gradient Pump; IPC: Intermittent pneumatic compression; NIRS: Near-infrared spectroscopy; O2Hb: Oxyhaemoglobin; PAD: Peripheral arterial disease; SFC: Sequential foot-calf device; TAV: Time average velocity; tHb: Total haemoglobin.

## Competing interests

The Authors declare that they have no competing interests.

Paolo Zamboni and Fabio Manfredini hold the intellectual property of the patent without any economic implication with companies.

## Authors’ contribution

FM, conceived and designed the study, analysed and interpreted the data, drafted the manuscript, and approved the final version of the manuscript; AMM, collected the data, analysed and interpreted the data, drafted the manuscript, and approved the final version of the manuscript; MF, collected the data, analysed and interpreted the data, and approved the final version of the manuscript; SM, collected the data, and approved the final version of the manuscript; NL, analysed and interpreted the data, and approved the final version of the manuscript; RM, analysed and interpreted the data, and approved the final version of the manuscript; FM, analysed and interpreted the data, and approved the final version of the manuscript; NB, analysed and interpreted the data, and approved the final version of the manuscript; PZ, conceived and designed the study, analysed and interpreted the data, drafted the manuscript, and approved the final version of the manuscript.

## Authors’ information

All the Authors participate to the research activity of the Vascular Diseases Center of the University of Ferrara, Italy, directed by Professor Paolo Zamboni. PZ an FM (Fabio Manfredini) has developed and patented the new IPC device here presented (London Equitable Ltd In Its Capacity As Trustee Of The Think Tank Trust [GB]. Device for pneumatic treatment of an inferior limb having peripheral arteriopathy problems. WO2010004592 (A1). 2010 January 14).

FM (Fabio Manfredini) is an assistant professor of the Department of Biomedical and Specialty Surgical Sciences - Section of Sport Sciences of the University of Ferrara, involved in exercise therapy for patients with peripheral arterial disease.

AMM is a postdoctoral medical doctor involved in researches on vascular diseases.

MF, SM and NL are sport sciences graduated involved in the current research during their PhD course.

RM is associate professor of Internal Medicine and the Director of the Operative Unit of Clinica Medica, S. Anna Hospital University of Ferrara.

NB is the Director of the Department of Rehabilitation Medicine, S. Anna Hospital University of Ferrara.

FM (Francesco Mascoli) is the Director of the Unit of Vascular and Endovascular Surgery, S. Anna Hospital University of Ferrara.

PZ is the Director of the Vascular Diseases Center of the University of Ferrara and past President of the International Society for Neurovascular Diseases.

## Pre-publication history

The pre-publication history for this paper can be accessed here:

http://www.biomedcentral.com/1471-2261/14/40/prepub
